# Role of cognitive reserve in ischemic stroke prognosis: A systematic review

**DOI:** 10.3389/fneur.2023.1100469

**Published:** 2023-02-22

**Authors:** Chunhua Tao, Yuan Yuan, Yijun Xu, Song Zhang, Zheng Wang, Sican Wang, Jingyan Liang, Yingge Wang

**Affiliations:** ^1^Department of Neurology, Affiliated Hospital of Yangzhou University, Yangzhou, China; ^2^School of Nursing and School of Public Health, Yangzhou University, Yangzhou, China; ^3^Division of Satoyama Nursing and Telecare, Nagano College of Nursing, Komagane, Japan; ^4^Department of the Advanced Biomedical Research, Interdisciplinary Graduate School of Medicine, University of Yamanashi, Chuo, Japan; ^5^Department of Biomedical Science and Institute of Bioscience and Biotechnology, Kangwon National University, Chuncheon-si, Gangwon-do, Republic of Korea; ^6^Department of Anatomy, Medical College, Yangzhou University, Yangzhou, China; ^7^Jiangsu Key Laboratory of Integrated Traditional Chinese and Western Medicine for Prevention and Treatment of Senile Diseases, Yangzhou University, Yangzhou, China

**Keywords:** cognitive reserve, ischemic stroke, mortality, functional outcome, systematic-analysis

## Abstract

**Objective:**

This systematic review was performed to identify the role of cognitive reserve (CR) proxies in the functional outcome and mortality prognostication of patients after acute ischemic stroke.

**Methods:**

PubMed, Embase, Web of Science, and Cochrane Library were comprehensively searched by two independent reviewers from their inception to 31 August 2022, with no restrictions on language. The reference lists of reviews or included articles were also searched. Cohort studies with a follow-up period of ≥3 months identifying the association between CR indicators and the post-stroke functional outcome and mortality were included. The outcome records for patients with hemorrhage and ischemic stroke not reported separately were excluded. The Quality In Prognosis Studies (QUIPS) tool was used to assess the quality of included studies.

**Results:**

Our search yielded 28 studies (*n* = 1,14,212) between 2004 and 2022, of which 14 were prospective cohort studies and 14 were retrospective cohort studies. The follow-up period ranged from 3 months to 36 years, and the mean or median age varied from 39.6 to 77.2 years. Of the 28 studies, 15 studies used the functional outcome as their primary outcome interest, and 11 of the 28 studies included the end-point interest of mortality after ischemic stroke. In addition, two of the 28 studies focused on the interest of functional outcomes and mortality. Among the included studies, CR proxies were measured by education, income, occupation, premorbid intelligence quotient, bilingualism, and socioeconomic status, respectively. The quality of the review studies was affected by low to high risk of bias.

**Conclusion:**

Based on the current literature, patients with ischemic stroke with higher CR proxies may have a lower risk of adverse outcomes. Further prospective studies involving a combination of CR proxies and residuals of fMRI measurements are warranted to determine the contribution of CR to the adverse outcome of ischemic stroke.

**Systematic review registration:**

PROSPERO, identifier CRD42022332810, https://www.crd.york.ac.uk/PROSPERO/.

## Introduction

It is well established that stroke is one of the leading causes of death and long-term disability worldwide among adults (1). Especially, older patients aged ≥75 years are at an increased risk of suffering from stroke during the last decades of their life (2), which imposes an enormous burden on global public health (3). Among stroke survivors, they were more likely to present significant deficits in multiple domains, including motor and cognitive impairment, disability, and psychological disorders (4, 5). Previous studies demonstrated that the first 3 months after ischemic stroke is a critical period of recovery, followed by a stable stage (6, 7). Although numerous studies have been conducted to predict adverse clinical and functional outcomes after ischemic stroke (8–10), further studies are essential to understand the underlying factors of inter-individual heterogeneity that contributed to unfavorable stroke outcomes. The term conserve reserve (CR) was theoretically constructed to explain the inter-individual discrepancies between the severity of brain pathology and clinical manifestations (11). A consensus was reached on the definition of CR in a recent whitepaper, defining CR as an active model of reserve acquired from various lifetime experiences (i.e., education attainment, intellectual activity, occupation history, and other environmental factors) *via* shaping the brain's network efficiency, processing capacity, and flexibility to protect against brain aging, pathology, or brain insult (12, 13). As measuring CR directly is full of challenges, sociobehavioral proxy indicators are commonly used to indirectly estimate CR, including education, occupation, leisure activities, premorbid intelligence quotient (IQ), socioeconomic status (SES), and/or bilingualism (14).

The concept of CR is well validated in patients with stroke as the study has found that the degree of cognitive impairment varied widely among individuals, despite comparable levels of pathology (13). An increasing number of studies have been carried out to examine the effects of potential CR proxies on the prediction and recovery of cognitive impairment after stroke (15–17). Previous studies provided some indication that CR may act as a crucial role in stroke recovery (17). Nevertheless, recent reviews or original studies focused only on the effect of educational attainment as an indicator of CR on post-stroke cognition, neglecting other functional stroke outcomes (i.e., disability, psychological disorders, and motor impairment) (16, 18). To the best of our knowledge, no systematic review or meta-analysis was conducted to methodically summarize the impact of CR sociobehavioral proxies on post-stroke functional outcomes and mortality.

To comprehensively assess the impact of CR sociobehavioral proxies on ischemic stroke outcomes, we performed a systematic review to identify the association of CR proxies with stroke outcomes, taking inter-individual variability into consideration.

## Methods

### Search strategy

The study was performed in conformity with the Preferred Reporting Items for Systematic Reviews and Meta-Analyses (PRISMA) guidelines (Supplementary Data Sheet 1). A systematic and comprehensive literature search was conducted in PubMed, Embase, Web of Science, and Cochrane Library, from their inception to 31 August 2022. We used two keywords, namely, “ischemic stroke” and “cognitive reserve” that were cross-searched by two independent reviewers. The phrase “cognitive reserve” as this term is sometimes used interchangeably with education, occupation, IQ, bilingualism, leisure activities, and socioeconomic status (14, 19, 20). In addition, our study was designed to focus on the prognosis of ischemic stroke. Keywords such as “prognosis” or “stroke outcome” were used for retrieval. No language limitations were used. The complete list of keywords for each literature search is available in Supplementary Data Sheet 2 (Table S1).

### Studies selection

Two authors (YY and XY) independently identified the article, abstract, and keywords of each article and evaluated the eligibility. Any discrepancies were discussed and resolved by a third referee (ZS). Studies that met the following criteria were included in this systematic analysis: (1) human study (participants age ≥18 years old); (2) cohort study (prospective cohort study or retrospective cohort study); (3) CR proxies as the exposure of interest (i.e., education, occupation, IQ, bilingualism, leisure activities, and socioeconomic status); (4) mortality and functional outcomes (i.e., post-stroke cognitive impairment or post-stroke depression) as the end-point of interest; (5) minimum follow-up period ≥3 months. The following studies were excluded: studies focusing on the transient ischemic attack (TIA) or with a combined record of patients with hemorrhagic and ischemic stroke, not first-ever patients with stroke, conference abstracts, letters, comments, editorials, and case reports. We also excluded systematic reviews and/or meta-analysis, but their reference lists were searched to identify primary studies.

### Data extraction

Data from the studies included were independently extracted by three authors (TC, WS, and WZ) through a standardized electronic form. We collected the following data elements for this study: study characteristics (first author, publication year, country, journal, and study design), demographic data (age and proportion of women), population recruitment interval, stroke types, the length of follow-up, the number of patients in the cohorts/number of participants with poor outcome (*n* total/*n* outcomes), cognitive reserve indicators, outcome definition and assessment, the type of statistical model, main findings, and the relationship between cognitive reserve and the outcome.

### Outcome definition

The prespecified primary outcome of interest was unfavorable functional outcomes, including post-stroke cognitive impairment or dementia, disability, and psychological dysfunction (depression or anxiety). Similarly, we considered the secondary outcome as death after a first-ever stroke. The modified Rankin Scale (mRS), the Barthel Index (BI), the Mini-Mental State Examination (MMSE), and the Hospital Anxiety and Depression Scale (HADS) were the common instruments to assess disability, motor impairment, cognitive dysfunction, and psychological disorders after ischemic stroke.

### Quality assessment

To critically appraise and evaluate the methodological quality of included studies, the Quality in Prognosis Studies (QUIPS) tool which is an optimal assessment tool was used, allowing an evaluation of risk bias and consideration of different CR proxies as prognostic factors (21). Accordingly, we evaluated the potential bias of each study in terms of study participation, study attrition, prognostic factor measurement, outcome measurement, study confounding, as well as statistical analysis and reporting. The outcomes were divided into low-, medium-, and high-risk biases.

## Results

### Literate search

**Figure 1:**
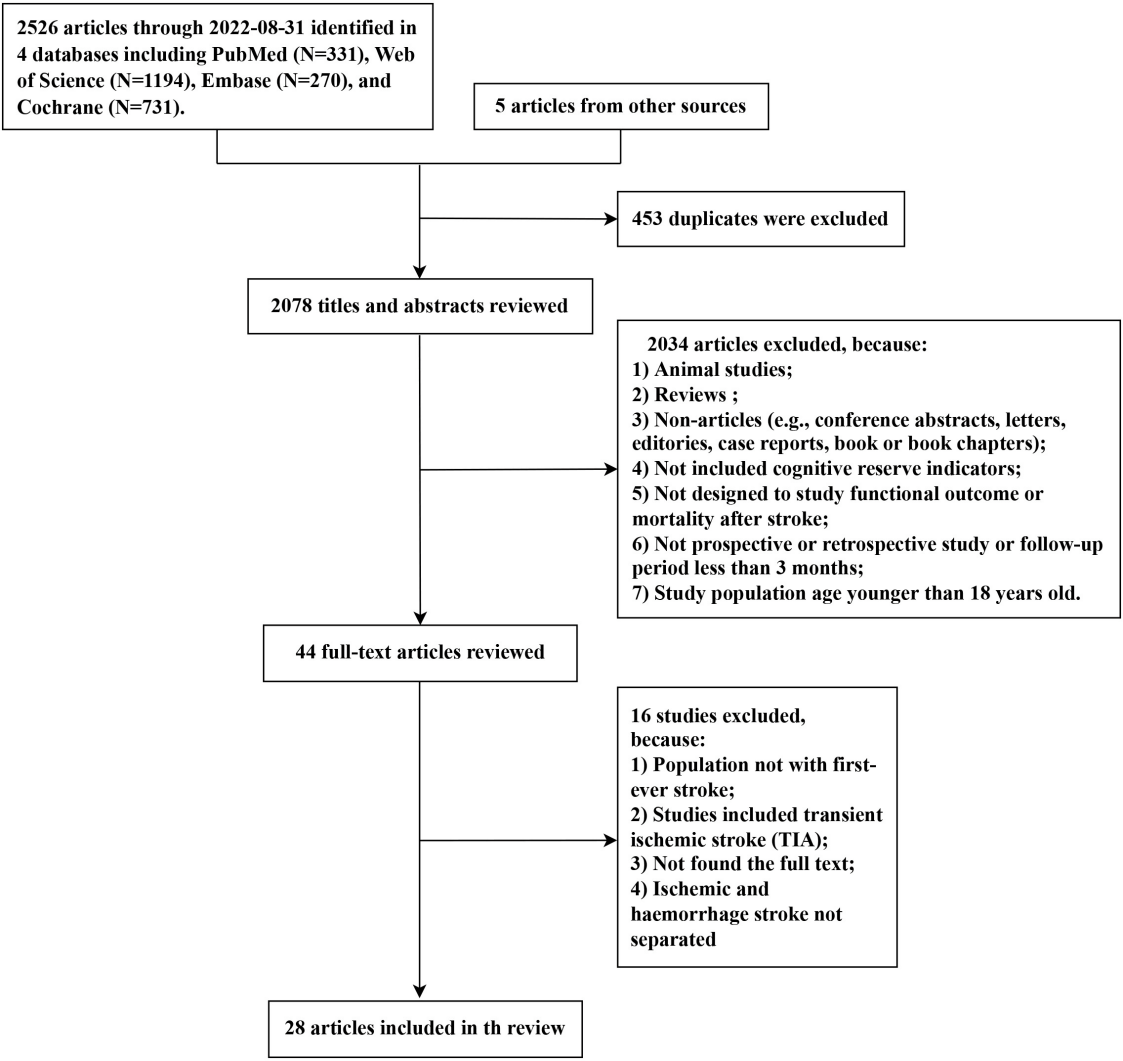
Flowchart of study selection.

The selection procedure for the systematic review is illustrated in Figure 1. The search yielded 2,526 articles from four databases (PubMed: 331, Web of Science: 1,194, Embase: 270, and Cochrane: 731). A total of five additional studies were identified from the references of relevant reviews. Among the 2,531 studies, we eliminated 453 duplicated articles and retained 44 articles for the full-text review after rigorously screening titles and abstracts. Ultimately, 28 articles met the eligibility criteria and were included in this systematic review. Given that the result data of meta-analyses demonstrated significant heterogeneity between studies, we attempted to present the synthesis of the studies in a narrative review format.

### Characteristics of included studies

The basic characteristics of the 28 included studies are summarized in Table 1. Studies in this review encompassed a total of 1,14,212 patients with stroke and were published between 2004 and 2022, with an average or median age varied from 39.6 to 77.2 years. The follow-up period ranged from 3 months to 36 years. A total of 12 studies were conducted in China (22–24, 35, 37, 38, 40, 42, 43, 45, 47, 48), three studies in the USA (30, 32, 49), two studies in the UK (33, 39), and one study each in Italy (25), Australia (26), Sweden (27), Germany (28), Finland (29), Brazil (31), India (34), Korea (36), Spain (41), France (44), and Czech Republic (46). A total of 15 studies (22, 24, 26, 28, 29, 32–34, 37, 39, 40, 42, 45–47, 49) involving 21,517 patients were pooled for an evaluation of functional outcome, eight of which (22, 26, 28, 39, 40, 42, 47, 49) adopted prospective designs. A total of 11 studies (23, 25, 27, 30, 31, 35, 36, 41, 43, 44) reported the outcome of mortality, of which four studies (23, 31, 43, 44) were designed as prospective studies, and the rest were retrospective studies. In addition, data from two prospective studies (29, 38) were pooled for an assessment of mortality and functional outcomes. Furthermore, the data of two studies (35, 37) were from the same retrospective cohort study but reported different outcomes of stroke, so we included them both. A total of 23 studies (22–24, 26, 28, 29, 32–35, 48) enrolled patients with first-episode ischemic stroke, and five studies (25, 27, 30, 31, 44) selected patients with ischemic and hemorrhagic stroke but reported separately. In addition, 16 studies (23, 25, 27, 28, 30, 32, 35–38, 41, 42, 44, 49) defined cognitive reserve indicators as socioeconomic status, eight studies (22, 24, 29, 31, 40, 43, 45, 47) used education attainment, two studies (26, 39) measured premorbid IQ, and one study each estimated bilingualism (34) and occupation (48).

**Table 1:** Characteristics of studies included in the systematic review.

**Authors, year, journal**	**Country**	**Study design**	**Patient recruitment**	**Stroke types**	**Population characteristics (mean age, % female)**	**Follow-up period**	***n* total/*n* outcomes**	**CR dimension**	**Outcome evaluation**	**Main findings**
Zhou et al. (22), *J Neurol*	China	Cohort, prospective	1999–2000	Ischemic stroke	73.8 years 46.6%	3 months	434/87	Education	Functional outcome (DSM-IV, NTB)	Low educational level was identified as independent predictors of dementia after ischemic stroke.
Zhou et al. (23), *BMC Public Health*	China	Cohort, prospective	1999–2002	Ischemic stroke	77.2 years 45.2%	3 years	806/166	SES (education, occupation, taxable income and housing space)	Mortality	Lower SES had a negative impact on the outcome of first-ever stroke.
Liu et al. (24), *Clin Neurol Neurosurg*	China	Cohort, retrospective	2001–2005	Ischemic stroke	40.5%	Mean: 22.98 months	434/190	Education	Functional outcome (mRS)	Lower educational level was associated with the poor functional outcome of ischemic stroke.
Cesaroni et al. (25), *Stroke*	Italy	Cohort, retrospective	2001–2004	Ischemic and hemorrhagic stroke	Ischemic stroke: 72 years 47.1% Hemorrhagic stroke: 67.7 years 47.9%	1 yer	7680/1147	SEP (education, occupation, home ownership, family composition and citizenship)	Mortality	There was no evidence of socioeconomic disparities in short-term or first-year fatality in either ischemic or hemorrhagic cases.
Withall et al. (26), *Aging Ment Health*	Australia	Cohort, prospective	1997–2000	Ischemic stroke	73.6 years 43.6%	15 months	168/94	Premorbid IQ (NART-R)	Functional outcome (MMSE, ADL, IADL)	A favorable outcome after stroke was found to have significantly higher premorbid IQ.
Toivanen et al. (27), *Scand J Public Health*	Sweden	Cohort, retrospective	1991–2002	Ischemic and hemorrhagic stroke	49.5% (for total cohort)	12 years	9262/1142	Income	Mortality	The risk of stroke mortality was the highest in the lowest income group, with a gradient for the intermediate groups.
Grube et al. (28), *Stroke*	Germany	Cohort, prospective	2010–2011	Ischemic stroke	40%	3 months	1688/219	SES (education)	Functional outcome (BI)	Patients with a lower education level had considerably lower rates of good functional outcomes after stroke.
Ojala-Oksala et al. (29), *Stroke*	Finland	Cohort, prospective	1993–2006	Ischemic stroke	72 years 49.9% (for total cohort)	Mean: 7.4 years	486/214	Education	Mortality cognitive function (NTB)	Educational history as a proxy indicator of cognitive reserve protected against deficits induced by acute stroke.
Brown et al. (30), *Neurology*	USA	Cohort, retrospective	1989–1990, 1992–1999	Ischemic and hemorrhagic stroke	74.5 years 60.5% (for total cohort)	1 year	806/276	NSES (*z*-scores of SES indicators: household income; value of housing units; education level; occupation)	Mortality	Living in a socioeconomically disadvantaged neighborhood was associated with higher mortality hazard at 1 year following an incident stroke.
Goulart et al. (31), *BMC Neurol*	Brazil	Cohort, prospective	2006–2010	Ischemic and hemorrhagic stroke	68 years 46.2% (for total cohort)	4 year	665/346	Education	Mortality	Lack of formal education was significant prognostic factors associated to higher mortality in patients with ischemic stroke during follow-up.
Bettger et al. (32), *BMC Public Health*	USA	Cohort, retrospective	2006	Ischemic stroke	69 years 52.4%	3 month	1965/679	SES (education, working status, household income)	Functional outcome (mRS)	Socioeconomic status was associated with disability following acute ischemic stroke.
Chen et al. (33), *Stroke*	UK	Cohort, retrospective	1995–2011	Ischemic stroke	47.4% (for total cohort)	3 year	2128/939	SED (IMD)	Functional outcome (BI)	SED was associated with short- and long-term functional impairment after stroke.
Alladi et al. (34), *Stroke*	India	Cohort, retrospective	2006–2013	Ischemic stroke	56.5 years 21.4%	2 year	608/415	Bilingualism	Functional outcome (ACE-R)	Bilingualism led to a better cognitive outcome after stroke, possibly by enhancing cognitive reserve.
Pan et al. (35), *Int J Stroke*	China	Cohort, retrospective	2007–2008	Ischemic stroke	65.5 years 38.2% (for total cohort)	1 year	12246/1540	SED (Education, occupation, income)	Mortality	SES was significantly associated with increased mortality in patients with ischemic stroke.
Shin et al. (36), *J Epidemiol*	Korea	Cohort, retrospective	2002–2013	Ischemic stroke	73%	3 year	37044/2334	Regional-level SES (Carstairs deprivation index score, individual income)	Mortality	Higher mortality among patients with stroke had low individual incomes and lived in high-SES regions.
Song et al. (37), *PLoS ONE*	China	Cohort, retrospective	2007–2008	Ischemic stroke	42.5%	3 month	11226/4721	SES (Education, occupation, monthly income)	Functional outcome (mRS)	People who were relatively more deprived in socioeconomic status suffered poorer outcomes after ischemic stroke.
Yan et al. (38), *Int J Med Sci*	China	Cohort, prospective	2012–2015	Ischemic stroke	65.9 years 48.4% (for total cohort)	Mean: 31.6 month	471/39	SES (Education, occupation, annual income and medical insurance) Neighborhood status	Functional outcome (mRS) Mortality	A lower personal SES as well as poorer neighborhood status may significantly increase risk for adverse clinical outcomes among patients with ischemic stroke.
Makin et al. (39), *Eur Stroke J*	UK	Cohort, prospective	2010–2012	Ischemic Stroke	66 years 41% (for total cohort)	1 year	151/29	premorbid IQ (NART) education	Functional outcome (ACE-R)	Premorbid IQ and education were stronger predictors of post-stroke cognition.
Ding et al. (40), *J Alzheimers Dis*	China	Cohort, prospective	2017–2018	Ischemic Stroke	64 years 33.8%	1 year	145/77	Education	Functional outcome (NTB, CDR)	A higher educational level indicated better cognitive reserve, which leads to a better favorable cognitive outcome after stroke.
Vivanco-Hidalgo et al. (41), *Stroke*	Spain	Cohort, retrospective	2015–2016	Ischemic Stroke	75 years 46.5% (for total cohort)	Mean: 18 month	16344/4249	SES (PCSA Index, drug dispensation)	Mortality	Individuals' socioeconomic status was associated with short- and long-term survival in patients with ischemic stroke.
Wang et al. (42), *Neurol Res*	China	Cohort, prospective	2004	Ischemic Stroke	69.9 years 35.9%	2 year	542/184	SES (education, income, caregiver and insurance)	Functional outcome (mRS)	Low income, family caregiver, and no insurance were significantly associated with the risk of poor prognosis of ischemic stroke. However, association between education and outcome of ischemic stroke was failed to find.
Che et al. (43), *J Am Heart Assoc*	China	Cohort, prospective	2009–2013	Ischemic Stroke	62 years 35.5% (for total cohort)	2 year	3861	Education	Mortality	Low education level was significantly associated with an increased risk of mortality after ischemic stroke.
Béjot et al. (44), *Eur J Neurol*	France	Cohort, prospective	2011–2014	Ischemic and hemorrhagic stroke	Ischemic stroke: 68 years 44% Hemorrhage stroke: 33.3%	1 year	1540/221	Social deprivation (EPICES score)	Mortality	Social deprivation was associated with delayed mortality in patients with ischemic stroke only. While in intracerebral hemorrhage, deprivation status was not associated with 12-month survival.
Dong et al. (45), *Aging*	China	Cohort, retrospective	2017–2018	Ischemic Stroke	64 years 32.2%	6 month	383/131	Education	Functional outcome (MoCA)	Education was found independently to predict post-stroke cognitive impairment.
Franc et al. (46), *Cent Eur J Public Health*	Czech Republic	Cohort, retrospective	2011–2020	Ischemic Stroke	39.6 years 45.1% (for total cohort)	3 month	297/60	SES (Education, marital status, income, occupation, and place of residence)	Functional outcome (mRS)	Patients with lower SES had poorer outcomes in comparison with those with higher SES.
Liu et al. (47), *Medicine*	China	Cohort, prospective	2014–2016	Ischemic Stroke	67.5 years 36.4% (for total cohort)	3 year	250/80	Education	Functional outcome (HADS)	The educational level independently predicted increased post-stroke anxiety or depression risk in patients with AIS.
Zhu et al. (48), *BMC Public Health*	China	Cohort, retrospective	2011–2013	Ischemic Stroke	63.4 years 32% (for total cohort)	1 year	1484/69	Occupation	Mortality	Mortality after ischemic stroke in the relationship between occupations was not demonstrated.
Ghoneem et al. (49), *JAMA Netw Open*	USA	Cohort, prospective	2009–2011	Ischemic Stroke	68.1 years 44.7% (for total cohort)	3 month	1098/NA	SES (Median household income, ADI)	Functional outcome (mRS)	Independent associations between socioeconomic status as well as post-stroke disability were found.

CR, cognitive reserve; DSM-IV, diagnostic and statistical manual of mental disorders, 4th edition; NTB, neuropsychological test battery, which was developed to make diagnosis of dementia, including mini-mental state examination (MMSE); ADL, activity of daily living; IADL, instrumental activity of daily living; POD, pfeiffer outpatient disability questionnaire; FOM, fuld object memory evaluation; RVR, rapid verbal retrieve; Wechsler Adult Intelligence Scale (DS and BD subtest) and Hamilton Depression Rating Scale; SES, socioeconomic status; mRS, modified rankin scale; SEP, socioeconomic position; IQ, intelligence quotient; NART-R, national adult reading test-revised; BI, barthel index; NSES, neighborhood socioeconomic status; SED, socioeconomic deprivation; IMD, index of multiple deprivation; ACE-R, addenbrooke's cognitive examination-revised; CDR, clinical dementia rating; PCSA Index, primary care service area socioeconomic index; EPICES, Evaluation de la Précarité et des Inégalités de santé dans les Centers d' Examen de santé; MoCA, montreal cognitive assessment; HADS, hospital anxiety and depression scale; ADI, area deprivation index.

### Quality assessment

**Figure 2:**
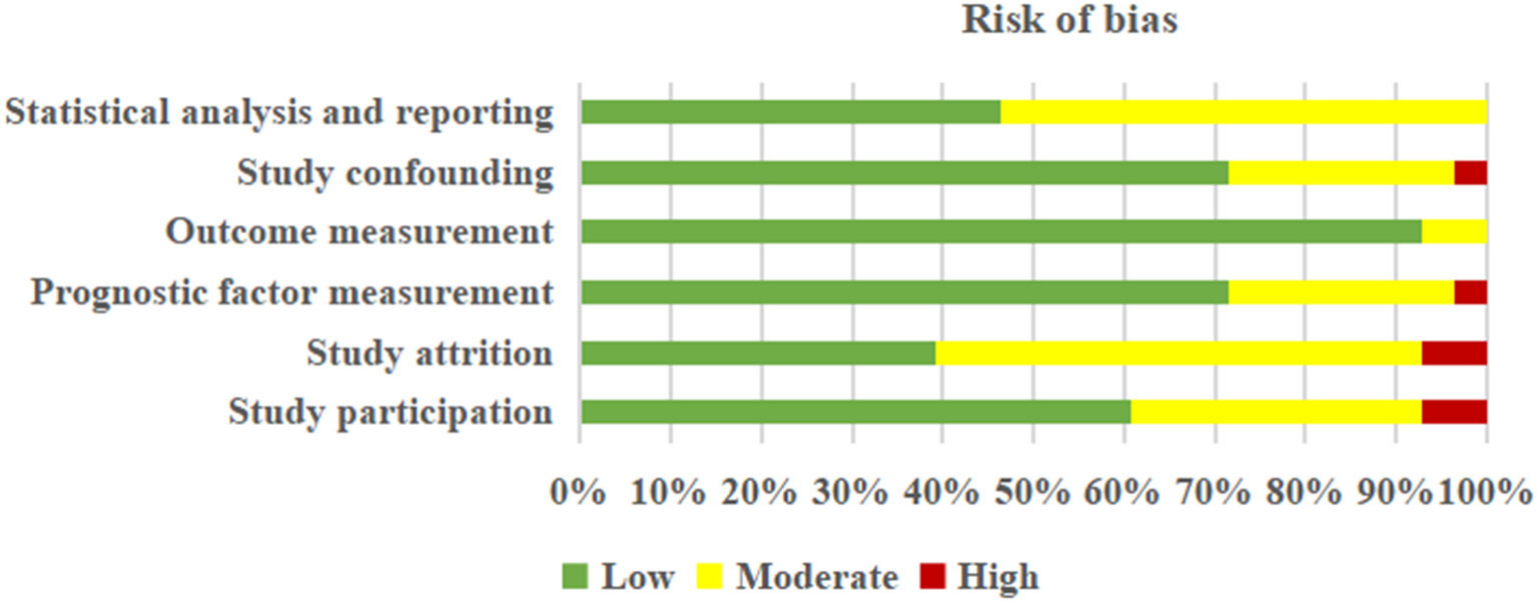
Summarized risk of bias in the 28 included studies according to the QUIPS criteria.

Detailed information about the judgment of each “risk of bias” domain is tabulated in Supplementary Data Sheet 2 (Table S2). Based on the QUIPS tool, no studies were excluded. In short, five (27, 29, 31, 32, 34) out of 28 studies were rated as having a high risk of bias, eight (22, 24, 26, 39, 45–48) as moderate, and 15 (23, 25, 28, 30, 33, 35–38, 40–44, 49) as low. Overall, the risk of six domains was judged to vary from low to high-risk bias, respectively, in “study participation” (low risk: 60.71%, moderate risk: 32.13%, and high risk: 7.14%), “study attrition” (low risk: 39.29%, moderate risk: 53.57%, and high risk: 7.14%), “prognosis factor measurement” (low risk: 71.43%, moderate risk: 25.00%, and high risk: 3.57%), “outcome measurement” (low risk: 92.86% and moderate risk: 7.14%), “study confounding” (low risk: 71.43%, moderate risk: 25.00%, and high risk: 3.57%), and “statistical analysis and reporting” (low risk: 46.43% and moderate risk: 53.57%) (Figure 2).

### Association of cognitive reserve with the functional outcome of ischemic stroke

A total of 17 studies reported the functional outcome at the end of follow-up, with the endpoint interest of cognitive dysfunction, disability, motor impairment, depression, or anxiety after ischemic stroke (22, 24, 26, 28, 29, 32–34, 37–40, 42, 45–47). A summary of the findings in the included literature that identified the association between CR and functional outcomes is demonstrated in Supplementary Data Sheet 2 (Table S3).

Out of the seven studies using the modified Rankin Scale (mRS), the association between post-stroke disability and cognitive reserve was identified (24, 32, 37, 38, 42, 46, 49). Liu et al. (24) adopted a multivariate logistic regression model and found a significant relationship between poor outcomes and lower educational levels. Among the included literature, the definition of SES varied widely. A total of two studies used a composition of education, occupation, and income (32, 37). Only one study additionally included medical insurance, neighborhood status (38), and another study included marital status and place of residence (46). Another study measured a combination of education, income, caregiver, and insurance (42), and one study evaluated median household income and the area deprivation index (ADI), which combined 17 weighted census indicators (e.g., measures of education, employment, housing quality, and poverty) (49). Regardless of the definition of SES and the type of regression model used, a lower SES significantly increased the risk of adverse clinical outcomes among patients with ischemic stroke.

A total of two studies used the Barthel Index (BI) to confirm the relationship between SES and motor impairment after ischemic stroke (28, 33). Grube et al. (28) defined SES measured by education as CR proxies and found that the lower the education attainment, the worse the functional outcome. In addition, the index of multiple socioeconomic deprivations based on patient postcodes as an SES indicator was found to be strongly associated with short- and long-term motor impairment after stroke (33). Both studies adopted the multivariate logistic regression model.

A total of six studies used various cognitive testing scales to examine the correlation between CR proxies and the post-stroke cognitive outcome (22, 29, 34, 39, 40, 45). The included studies most frequently used education level as CR indicators (*n* = 4) and found that the higher the education level, the better the cognitive outcome (22, 29, 40, 45). Among the four studies, three of them adopted the logistic regression model (22), binomial logistic regression model and multivariate logistic regression model respectively, whereas the other study did not provide the method used (29). Using bilingualism as an indicator of CR, it is found that bilingualism contributes to better cognitive outcomes, based on the logistic regression model (34). Makin et al. (39) defined composition of premorbid IQ and education as CR indicators and found that both of them were stronger predictors of post-stroke cognition through the logistic and linear regression model.

A study, using a combination index of the mini-mental state examination (MMSE) scores, the activity of daily living (ADL) scores, and the instrument activity of daily living (IADL) scores, identified the association between premorbid IQ and the clinical outcome after ischemic stroke. Based on the multivariate logistic regression model, the study found that better outcomes after stroke had significantly higher premorbid IQ (26).

### Association of cognitive reserve with the mortality of ischemic stroke

A total of 13 studies reported the mortality outcome at the end of the follow-up (23, 25, 27, 29–31, 35, 36, 38, 41, 43, 44, 48). A total of three studies defined education as CR proxies and found that educational history was associated with lower mortality after ischemic stroke through the Cox regression model and Cox proportional hazard model (31, 43). One each study regarded income or occupation as CR indicators, and both of them used the Cox proportional hazard model, but the results were different (27, 48). The association of lower income with the increase in the risk of stroke mortality was found in the study by Toivanen et al. (27). Nevertheless, Zhu et al. (48) failed to find any relationship between occupation and mortality after ischemic stroke. A total of eight studies assessed whether SES as a proxy for CR was significantly associated with mortality after stroke, based on the various definitions of SES (23, 25, 30, 35, 36, 38, 41, 44). Pan et al. (35) defined a composition indicator of education, occupation, and income as SES, and Zhou et al. (23) additionally included housing space as one of the indicators. Brown et al. (30) included the value of the housing unit, and Yan et al. (38) also included medical insurance. Similarly, it was concluded that lower SES had a negative impact on the outcome of first-ever stroke. Moreover, Shin et al. (36) calculated the Carstairs deprivation index score and individual income as SES indicators and found that low individual incomes and living in high-SES regions enhanced higher mortality among patients with stroke. Vivanco-Hidalgo et al. (41) considered the Primary Care Service Area Socioeconomic (PCAS) index scores and drug dispensation as SES indicators, finding that an individual's SES was related to survival in patients with ischemic stroke. SES measured by socioeconomic deprivation was strongly correlated with delayed mortality in patients with ischemic stroke (44). Of the eight articles, three studies adopted Cox proportional hazard models (23, 30, 36), and each opted for the logistic regression model (25), multivariate-adjusted logistic regression model (35), and mixed-effects logistic and survival model (41) and multivariate Cox model (38, 44), respectively. Supplementary Data Sheet 2 (Table S4) shows detailed information on the association between CR and mortality after stroke.

## Discussion

Eventually, this systematic review included 14 prospective cohort studies and 14 retrospective cohort studies to identify the association between CR proxies and the prognosis of ischemic stroke, as measured by education, income, occupation, premorbid IQ, bilingualism, and SES as CR proxies. The results reveal that lower scores on CR proxies may have an important potential role in predicting motor and cognitive impairment, disability, psychological disorders, and mortality after ischemic stroke.

Consistent with our findings, several previous studies that did not meet the eligibility criteria for the current systematic review also reported that CR characterized by neural reserve and compensation might partially address the gap in the heterogeneity of stroke injury and recovery (11, 15). More specifically, neural networks protect against neurological damage in ischemic stroke by spontaneously utilizing, optimizing, strengthening existing effective cognitive process, or recruiting alternate pathways (11, 50). However, the exact mechanisms of CR remain uncertain. Although the concept of CR is a theoretical construct, various methods attempted to operationalize and measure CR. Education, occupation, leisure activity, and premorbid IQ were commonly used to measure CR indirectly (14, 51). Among the limited methodological studies, most of them investigated only a single CR proxy to reflect the CR level for the feasibility and expedient of operationalization. Furthermore, some of the included studies used different evaluation methods for the indicator itself (e.g., education and SES), which may make a difference in the results of CR. Hence, we should possess a cautious attitude toward these results as CR is constructed by multidimensional components.

A previous study ascertaining the prognostic role of CR in stroke-induced functional impairment and mortality is limited. Low educational attainment was shown to be associated with disability, mortality, worse cognitive function, and higher risk of depression or anxiety in the stable stroke phase (24, 43, 44, 47). Shin et al. (16) found that a higher educational level can predict the recovery of active and stable phases after the stroke onset. Educational history as a proxy indicator of CR is significantly associated with post-stroke cognitive deficits, dementia, and long-term survival, independent of age, gender, stroke severity, and white matter lesions (WML) in mild/moderate ischemic patients with stroke (52). Patients with a higher educational level may have more synapses, larger brains, or more efficient brain networks to tolerate more pathology until reaching a critical threshold and presenting a cognitive deficit later (13, 53).

Heretofore, the evidence that occupation complexity, bilingualism, income, and premorbid IQ as proxies for CR are correlated with the long-term outcome of ischemic stroke is relatively limited. The low complexity of occupation was found to be associated with a high risk of cognitive impairment and decreased the speed of cognitive recovery (16). Alladi et al. (34) found that bilingualism served as a protective role in the development of post-stroke cognitive impairment, independent of age or vascular risk factors. Compared with the highest income group, patients with the lowest income have a considerably higher risk of stroke mortality, with a gradient for the intermediate groups (27). Premorbid IQ as a proxy indicator of CR was found to be a stronger predictor of the long-term post-stroke cognition outcome and late-life depression and dementia (26, 39). The favorable impacts of occupation, bilingualism, income, and premorbid IQ on the cognitive function and mortality of stroke outcomes can be explained by CR theory. Patients with a higher CR level might be more capable of resisting stroke damage by recruiting alternative functional centers and providing easier and faster compensation (15, 54).

In the past decades, there has been a widespread interest in exploring the relationship between SES as a proxy indicator of CR and stroke outcomes. Socioeconomic status is a complex conception that combined economics and sociology to reflect an individual's or family's position, commonly based on education, occupation, and income (55). Studies found that the frequency of motor impairment, mortality, and disability was lower than that in patients with a higher SES level (33, 44, 49, 56). However, the association between SES and stroke mortality was inconsistent. Several studies yielded no correlation between SES and stroke mortality, which may be due to differences in the selected SES indicators or regions (57). To provide robust evidence on the relationship between SES and stroke outcome, Wang et al. (55) summarized the evidence and found that patients with a low SES level had a higher risk of stroke mortality despite the heterogeneity of each SES indicator. The underlying mechanisms between SES and the increased risk of stroke mortality remain unclear. The long-term outcome of stroke may depend on the differences in inter-individual SES level in the initial stroke severity, independent of treatments and symptom duration (49). SES, as a significant component of CR, is influenced by lifetime experiences and decreases structural brain changes through shaping network efficiency, processing capacity, and flexibility (13).

While the exact mechanism of CR on the prognostic performance of long-term outcomes after ischemic stroke has not been clarified clearly, the impact of CR on inferior outcomes might comprise the following underlying mechanisms (15). First, patients with a higher CR level may tolerate more pathology until reaching a critical threshold manifested by cognitive deficits despite comparable stroke severity (58). Second, based on the CR theory, dendritic or plasticity was fostered to improve network efficiency and capacity to resist brain pathology after ischemic stroke (53). Third, higher CR was linked to recruiting alternative neural networks to provide faster compensation for brain injury (54). Overall, CR is a dynamic and modifiable reserve model affected by lifetime intellectual activities (12). In the present studies, the residual method was proposed to directly measure CR, which was calculated through the regression model combined with functional imaging results (12, 59).

Our systematic review reveals that cognitive reserve played a significant role in the prediction of stroke-related impairment and recovery, which resolves a major clinical challenge (60). To reduce the global burden of post-stroke disability and provide precise health promotion or prevention, cognitive reserve as a modifiable conception deserves further investigation.

## Strengths and limitations

To the best of our knowledge, this systematic review is the first attempt to comprehensively provide a summary impact of CR sociobehavioral proxies on the prognosis of patients with ischemic stroke. Nevertheless, we acknowledge that our study has several limitations. First, this review only considered sociobehavioral proxies as CR indicators, lacking other potential direct measurements of CR, and most studies merely used a single proxy. Second, given the heterogeneity of CR proxies, follow-up period, population, and stroke outcome, we failed to quantitatively summarize the data and have to perform a descriptive overview. Third, only longitudinal studies were included in this systematic review, and other potentially relevant randomized controlled trials may provide stronger evidence to reveal the underlying mechanism of CR and stroke outcomes. Finally, although some of the included studies were rated as having moderate to high-risk bias according to the QUIPS tool, we made the decision to retain these studies as the difficulty of avoiding bias in literature reviews, indicating that the results should be illustrated with caution.

## Conclusion

Our results provide evidence that lower CR proxies may have a significant association with unfavorable outcomes after ischemic stroke. However, given the limitations of this review, the results of this study ought to be treated cautiously. Accordingly, further prospective studies measuring CR with a multidimensional approach are warranted to develop a deeper understanding of the underlying neural mechanism of CR and its contribution to the prognosis of ischemic stroke.

## Data availability statement

The original contributions presented in the study are included in the article/Supplementary material, further inquiries can be directed to the corresponding author.

## Author contributions

JL and YW designed the study and search strategy. YY, YX, and SZ performed the literature search and assessment. CT, SW, and ZW contributed to data extraction. CT wrote the first draft of the manuscript, and all authors provided critical revision and approved the final version.
