# Supplementary material for: Role of cognitive reserve in ischemic stroke prognosis: A systematic review
Source: Front Neurol. 2023 Feb 22;14:1100469. doi: 10.3389/fneur.2023.1100469 (PMC9992812; doi:10.3389/fneur.2023.1100469)
Supplement: Supplementary file 2 [file Data_Sheet_2.pdf]

Table S1. Search Strategy and Results from Databases

Database	Number of Results	Search Terms
PubMed	1026	((Cognitive Reserve[MeSH Terms]) OR (cognitive reserve*[Title/Abstract]) OR (cognitive resilience[Title/Abstract]) OR (brain resilience[Title/Abstract]) OR (cognitive capacity[Title/Abstract]) OR (education[Title/Abstract]) OR (occupation[Title/Abstract]) OR (vocation[Title/Abstract]) OR (leisure activit*[Title/Abstract]) OR (bilingual*[Title/Abstract]) OR (IQ[Title/Abstract]) OR (intelligence quotient[Title/Abstract]) OR (multilingualism[Title/Abstract]) OR (SES[Title/Abstract]) OR (socioeconomic status[Title/Abstract])) AND ((Ischemic Stroke[MeSH Terms]) OR (ischaemic stroke[Title/Abstract]) OR (ischemic stroke[Title/Abstract]) OR (cerebrovascular accident[Title/Abstract]) OR (brain ischemia[Title/Abstract]) OR (brain infarction[Title/Abstract]) OR (cerebral ischemia[Title/Abstract]) OR (cerebral infarction[Title/Abstract]) OR (ischemic brain[Title/Abstract]) OR (cerebrovascular apoplexy[Title/Abstract]) OR (brain vascular accident[Title/Abstract]) OR (cerebrovascular stroke[Title/Abstract])) AND ((Prognosis[MeSH Terms]) OR (prognos*[Title/Abstract]) OR (predict*[Title/Abstract]) OR (mortality[Title/Abstract]) OR (death[Title/Abstract]) OR (disability[Title/Abstract]) OR (stroke outcome*[Title/Abstract]) OR (functional outcome*[Title/Abstract]) OR (functional status[Title/Abstract]) OR (modified Rankin Scale[Title/Abstract]) OR (mRS[Title/Abstract]) OR (NHISS[Title/Abstract])) AND ((cohort studies[MeSH Terms]) OR (cohort analysis[Title/Abstract]) OR (prospective study[Title/Abstract]) OR (follow-up studies[Title/Abstract]) OR (longitudinal studies[Title/Abstract]))
EMBASE	1772	('cognitive reserve'/exp OR ('cognitive reserve*':ab,ti OR 'cognitive resilience':ab,ti OR 'brain resilience':ab,ti OR 'cognitive capacity':ab,ti OR 'education':ab,ti OR 'occupation':ab,ti OR 'vocation':ab,ti OR 'leisure activit*':ab,ti OR 'bilingual*':ab,ti OR 'multilingualism':ab,ti OR 'IQ':ab,ti OR 'intelligence quotient':ab,ti OR 'SES':ab,ti OR 'socioeconomic status':ab,ti)) AND ('ischemic stroke'/exp OR ('ischaemic stroke':ab,ti OR 'ischemic stroke':ab,ti OR 'cerebrovascular accident':ab,ti OR 'brain ischemia':ab,ti OR 'brain infarction':ab,ti OR 'cerebral ischemia':ab,ti OR 'cerebral infarction':ab,ti OR 'ischemic brain':ab,ti OR 'cerebrovascular apoplexy':ab,ti OR 'brain vascular accident':ab,ti OR 'cerebrovascular stroke':ab,ti)) AND ('prognosis'/exp OR ('prognos*':ab,ti OR 'predict*':ab,ti OR 'mortality':ab,ti OR 'death':ab,ti OR 'disability':ab,ti OR 'stroke outcome*':ab,ti OR 'functional outcome*':ab,ti OR 'functional status':ab,ti OR 'modified Rankin Scale':ab,ti OR 'mRS':ab,ti OR 'NHISS':ab,ti)) AND ('cohort studies'/exp OR ('cohort stud*':ab,ti OR 'cohort analysis':ab,ti OR 'prospective study':ab,ti OR 'follow-up studies':ab,ti OR 'longitudinal study':ab,ti))
Cochrane	1871	([Cognitive Reserve] explode all trees OR Cognitive Reserve* OR Cognitive Resilience OR Brain Resilience OR Cognitive Capacity OR Education OR Occupation OR Vocation OR Leisure Activit* OR Bilingual* OR Multilingualism OR IQ OR Intelligence Quotient OR SES OR Socioeconomic Status):ti,ab,kw (Word variations have been searched) AND ([Ischemic Stroke] explode all trees OR Ischemic Stroke OR Ischaemic Stroke OR Cerebrovascular Accident OR Brain Ischemia OR Brain Infarction OR Cerebral Ischemia OR Cerebral Infarction OR Ischemic Brain OR

		Cerebrovascular Apoplexy OR Brain Vascular Accident OR Cerebrovascular Stroke):ti,ab,kw (Word variations have been searched) AND ([Prognosis] explode all trees OR Prognos* OR Predict* OR Mortality OR Death OR Disability OR Stroke Outcome* OR Functional Outcome* OR Functional Status OR modified Rankin Scale OR mRS OR NHISS):ti,ab,kw (Word variations have been searched) AND ([Cohort Studies] explode all trees OR Cohort Stud* OR Cohort Analysis OR Prospective Study OR Follow-up Studies OR Longitudinal Studies):ti,ab,kw (Word variations have been searched)
Web of science	1150	TS=((cognitive reserve OR cognitive reserve* OR cognitive resilience OR brain resilience OR cognitive capacity OR education OR occupation OR vocation OR leisure activit* OR bilingual* OR multilingualism OR IQ OR intelligence quotient OR SES OR socioeconomic status) AND (ischemic stroke OR ischaemic stroke OR cerebrovascular accident OR brain ischemia OR brain infarction OR cerebral ischemia OR cerebral infarction OR ischemic brain OR cerebrovascular apoplexy OR brain vascular accident OR cerebrovascular stroke) AND (prognosis OR prognos* OR predict* OR mortality OR death OR disability OR stroke outcome* OR functional outcome* OR functional status OR modified Rankin Scale OR mRS OR NHISS) AND (cohort studies OR prospective study OR cohort analysis OR follow-up studies OR longitudinal studies))

Table S2. Risk of Bias Assessment of Studies Included in Systematic-analysis According to QUIPS Tool

Authors, Year; (Study)	Study Population	Study Attrition	Prognostic Factor Measurement	Outcome Measurement	Confounding Measurement and Account	Analysis and Reporting	Overall Estimate
Zhou et al., 2004	Moderate	Low	Moderate	Low	Low	Moderate	Moderate
Zhou et al., 2006; (Nanjing Stroke Registry)	Low	Low	Low	Low	Low	Moderate	Low
Liu et al., 2007	Low	Low	Moderate	Low	Low	Moderate	Moderate
Withall et al., 2009	Low	Low	Moderate	Low	Low	Moderate	Moderate
Cesaroni et al., 2009; (Regional Cause of Death Registry)	Low	Low	Low	Low	Moderate	Moderate	Low
Toivanen et al., 2011; (Stockholm Karolinska Institute)	Moderate	Moderate	Low	Low	High	Moderate	High
Ojala-Oksala et al., 2012; (SAM)	Low	High	Moderate	Low	Moderate	Moderate	High
Grube et al., 2012; (Berlin Stroke Register)	Low	Moderate	Low	Low	Low	Moderate	Low
Goulart et al., 2013; (The EMMA Study)	Moderate	High	High	Low	Low	Moderate	High
Brown et al., 2013; (Cardiovascular Health Study)	Moderate	Moderate	Low	Low	Low	Low	Low
Bettger et al., 2014; (AVAIL, GWTG-Stroke)	High	Moderate	Low	Low	Moderate	Low	High
Chen et al., 2015; (SLSR)	Moderate	Low	Low	Low	Low	Low	Low
Alladi et al., 2016; (Nizam Stroke Registry)	High	Moderate	Low	Low	Moderate	Moderate	High
Pan et al., 2016; (CNSR)	Moderate	Low	Low	Low	Low	Low	Low
Shin et al., 2017; (KNHI)	Low	Moderate	Low	Moderate	Low	Low	Low
Yan et al., 2017	Low	Moderate	Low	Low	Low	Low	Low

Song et al., 2017; (CNSR)	Moderate	Low	Low	Low	Low	Low	Low
Makin et al., 2018	Moderate	Low	Low	Low	Moderate	Moderate	Moderate
Vivanco-Hidalgo et al., 2019; (Catalan Central Registry)	Low	Moderate	Low	Low	Low	Low	Low
Ding et al., 2019	Low	Low	Moderate	Low	Low	Low	Low
Wang et al., 2019	Low	Low	Low	Low	Low	Moderate	Low
Che et al., 2020; (CATIS)	Low	Moderate	Low	Low	Low	Low	Low
Béjot et al., 2021	Low	Moderate	Low	Low	Low	Low	Low
Dong et al., 2021	Moderate	Moderate	Moderate	Low	Moderate	Low	Moderate
Franc et al., 2021	Low	Moderate	Low	Low	Moderate	Moderate	Moderate
Liu et al., 2021	Low	Moderate	Moderate	Moderate	Low	Moderate	Moderate
Zhu et al., 2021; (IIPAIS)	Low	Moderate	Low	Low	Low	Moderate	Moderate
Ghoneem et al., 2022	Low	Moderate	Low	Low	Low	Low	Low

SAM, Stroke Aging Memory; The EMMA Study, The Study of Stroke Mortality and Morbidity; AVAIL, Adherence eValuation After Ischemic stroke-Longitudinal study; GWTG-Stroke, Get With The Guidelines-Stroke program; SLSR, South London Stroke Register; CNSR, China National Stroke Registry; KNHI, Korean National Health Insurance cohort; CATIS, China Antihypertensive Trial in Acute Ischemic Stroke; IIPAIS study, Infectious Factors, Inflammatory Markers and Prognosis of Acute Ischemic Stroke.

Table S3. Results of Studies with Functional Outcome After Stroke as the Primary Outcome

Authors, Year, Journal	Title	CR Proxies	Outcome Definition	Type of Regression Model Used	Association Between CR and Outcome
Zhou et al, 2004, *J Neurol*	Study on frequency and predictors of dementia after ischemic stroke: The Chongqing Stroke Study	Education (Illiterate, 1-6y, 7-9y, ≥10y)	Senior neurologists and psychiatrists made clinical diagnosis of dementia according to DSM-IV and NTB 3 month after stroke.	Logistic regression	Multivariate analyses demonstrated that low educational level (OR = 1.806, 95%CI: 1.024-3.186) was associated with PSD.
Liu et al, 2007, *Clin Neurol Neurosurg*	Prediction of functional outcome of ischemic stroke patients in northwest China	Education (Illiterate, elementary, junior, senior, university)	Disability was assessed according to mRS and categorized as good outcome (score 0-2) or poor outcome (score 3-6).	Multivariate logistic regression	The poor outcome was associated with lower educational level (OR = 0.686, 95%CI: 0.570-0.825).
Withall et al, 2009, *Aging Ment Health*	Who does well after a stroke? The Sydney Stroke Study	premorbid IQ (NART-R)	A favourable outcome was defined as having an MMSE score ≥28/30 in addition to an ADL+IADL score equal to 14/14 at the follow-up assessment.	Multivariate logistic regression	premorbid IQ (OR = 1.07, 95%CI: 1.01-1.14) served as an predictor of favourable outcome following stroke.
Grube et al, 2012, *Stroke*	Association between socioeconomic status and functional impairment 3 months after ischemic stroke: The Berlin Stroke Register	Education was used as an indicator of SES. (No completed education; basic or secondary education and no vocational training; basic or secondary education and vocational training; high school degree; and college or	Functional outcome was grouped into 3 categories: poor (BI, 0-70); moderate (BI, 75-95); and good (BI, 100).	Multivariate logistic regression	A higher probability of good outcome was observed in patients with college or university degree (OR = 2.18, 95%CI: 1.39-3.42) compared with patients with no completed education.

		university degree)			
Ojala-Oksala et al, 2012, *Stroke*	Educational history is an independent predictor of cognitive deficits and long-term survival in postacute patients with mild to moderate ischemic stroke	Education (0-6y, 7-9y, ⩾10y)	Patients were assessed the following domains by neuropsychological test: global cognitive function, executive functions, memory functions, language, visuospatial and constructional abilities.	Not provided	Educational history was independently associated with less memory impairment (OR = 0.67, $P<0.01$), aphasia (OR = 0.69, $P<0.05$), visuospatial and constructive deficits (OR = 0.70, $P<0.05$), MMSE score <25 (OR = 0.53, $P<0.0001$), and dementia (OR = 0.66, $P<0.01$).
Bettger et al, 2014, *BMC Public Health*	The association between socioeconomic status and disability after stroke: Findings from the Adherence eValuation After Ischemic stroke Longitudinal (AVAIL) registry	Low SES was defined as completing a high school degree or less; retired, disabled and not-working, or unemployed/homemakers; and income only somewhat or not at all met their basic needs	A mRS score of 3-5 was defined as post-stroke disability.	Multivariate logistic regression	Stroke survivors who were unemployed or homemakers (OR = 3.19, 95%CI: 2.02-5.02) disabled and not-working (OR = 2.46, 95%CI: 1.58-3.83) retired (OR = 1.87, 95%CI: 1.34-2.60) less educated (OR = 1.44, 95%CI: 1.12-1.85) or reported to have inadequate income prior to their stroke (OR = 1.91, 95%CI: 1.56-2.35) had a significantly higher odds of post-stroke disability.
Chen et al, 2015, *Stroke*	Association between socioeconomic deprivation and functional impairment after stroke: The South London Stroke Register	IMD was calculated to measure SED, covering 7 dimensions as follows: income; employment; health and disability; education, skills, and training; barriers to housing and services; living	BI was used to assess the functional impairment after stroke, which was grouped to <15 (severe/moderate disability), 15 to 19 (mild disability), and 20 (independent).	Multivariate -adjusted logistic regression	Compared with the first quartile of index of multiple deprivation (the least deprived), multivariate-adjusted odds ratios for functional impairment in patients with the second, third, and fourth quartiles were 1.29 (95%CI:

		environment; and crime.			0.94-1.76), 1.33 (0.97-1.82), and 1.78 (1.31-2.43), overall *P*=0.004.
Alladi et al, 2016, *Stroke*	Impact of bilingualism on cognitive outcome after stroke	Bilingualism was defined as the ability to communicate in 2 or more languages in interaction with other speakers of these same languages	Scores of ACE-R were classified into the following diagnostic groups: VaD, vascular mild cognitive impairment, aphasia, and strokes with normal cognition.	Logistic regression	Bilingualism was found to be an independent predictor of poststroke cognitive impairment (OR = 2.184, 95%CI: 1.379-3.458).
Song et al, 2017, *PLoS One*	Is there a correlation between socioeconomic disparity and functional outcome after acute ischemic stroke?	The patients with <6 years education, no job or manual workers, and individual income ≤$160 per month were defined as SED.	The mRS at 3 months after stroke was recorded to evaluate functional outcome, which is scaled to 6 points ranging from 0 (no symptoms) to 5 (severe disability).	Multiple and multinomial logistic regression	Compared to patients with educational level of ≥6 years and non-manual laboring, those <6 years and manual laboring tended to have higher mRS score (*P*<0.001). Multinomial adjusted odds ratios (ORs) of outcome in manual workers were significantly increased (ORs from1.38 to 1.87), but OR in patients with less income was not significant.
Yan et al, 2017, *Int J Med Sci*	The influence of individual socioeconomic status on the clinical outcomes in ischemic stroke patients with different neighborhood status in Shanghai, China	Education, occupation, annual income, and medical insurance as indicators of SES were categorized to five groups from low to high level, for which a gradually increasing score (0-4) was assigned and the final summed score of each factor represented the	A mRS score of 3-5 was considered as post-stroke disability.	Multivariate Cox regression	Both individual SES (HR = 0.767, 95% CI: 0.623-0.944; *P* = 0.012) and neighborhood status (HR = 0.730, 95% CI: 0.582-0.916; *P* = 0.007) are independently associated with the clinical outcomes in ischemic stroke patients.

		individual SES.			
Makin et al, 2018, *Eur Stroke J*	The impact of early-life intelligence quotient on post stroke cognitive impairment	NART was used to evaluate premorbid IQ and collected education attainment.	Cognitive impairment was defined as an ACE-R score <82 (sensitivity of 84%, specificity of 100% for dementia).	Logistic and linear regression	Lower NART score predicted one-year cognitive impairment (OR = 0.91, 95%CI: 0.87-0.95).
Ding et al, 2019, *J Alzheimers Dis*	Predictors of cognitive impairment after stroke: a prospective stroke cohort study	Education (0 y, 1-6 y, ≥7 y)	PSCI was identified as both MMSE and MoCA scores were lower than the cut-off values or NTB with more than one affected cognitive domain and CDR rating was greater than 0 points. PSD diagnosis was made according to DSM-IV.	Binomial logistic regression	Years of education was independently associated with 6 – 12month PSCI (β = –0.346, OR = 0.707, 95%CI: 0.607-0.824)
Wang et al, 2019, *Neurol Res*	Association between socioeconomic status and prognosis after ischemic stroke in South China	Education, monthly income, caregiver, and insurance were considered as indicators of SES. (Education: primary or below, junior, senior, university or higher; Monthly income: <$301, ≥$301; Caregiver: family caregiver, health-care assistant, and no caregiver; Insurance: no insurance, FMC, medical insurance)	Participants with an mRS score of 3-6 had a poor prognosis after stroke.	Multivariate and binomial logistic regression	Odds ratio for poor prognosis in patients with low income was 1.84 (95%CI: 1.05-3.22), family caregiver 3.19 (95%CI: 1.05-9.70), and no insurance 1.68 (95%CI: 1.02-2.77).
Dong et al, 2021,	Development and validation	Education	A patient with a 6-month MoCA	Multivariate	More than 9 years of educational level

Aging	of a clinical model (DREAM-LDL) for post-stroke cognitive impairment at 6 months	(<5 y, 5-9 y, 9-12 y, >12 y)	score <22 was diagnosed as PSCI.	logistic regression	was associated with better cognitive function after stroke (OR = 0.85, 95%CI: 0.63-1.14).
Franc et al, 2021, *Cent Eur J Public Health*	Socioeconomic status and lifestyle in young ischemic stroke patients: a possible relationship to stroke recovery and risk of recurrent Event	The assessment of SES involved level of education, income, occupation, marital status, and place of residence.	An excellent 3-month clinical outcome was defined as mRS score 0-1.	Not provided	Patients with MRS 0-1 did not differ in SES except having more frequently university education (21.1% vs. 3.3%, $p = 0.001$).
Liu et al, 2021, *Medicine*	Longitudinal assessment of anxiety/depression rates and their related predictive factors in acute ischemic stroke patients: A 36-month follow-up study	Education duration	HADS-A score ≥8 was defined as anxiety, and HADS-D score ≥8 was defined as depression.	Multivariate logistic regression	Longer education duration independently predicted raised anxiety and depression risk at 12month after stroke.
Ghoneem et al, 2022, *JAMA Netw Open*	Association of socioeconomic status and infarct volume with functional outcome in patients with ischemic stroke	The primary SES measure was local median household income using zip codes and the secondary SES measure was ADI.	Assessment of long-term disability was performed at a mean (SD) of 90 (15) days using the mRS score.	Linear regression	Income was inversely associated with long-term disability (standardized β = –0.092, 95% CI: –0.149-–0.035]; $P = 0.001$).

DSM-IV, Diagnostic and Statistical Manual of Mental Disorders, 4th edition; NTB, Neuropsychological Test Battery, which was developed to make diagnosis of dementia, including Mini-Mental State Examination (MMSE), Activity of Daily Living (ADL), Instrumental Activity of Daily Living (IADL), Pfeiffer Outpatient Disability Questionnaire (POD), Fuld Object Memory Evaluation (FOM), Rapid Verbal Retrieve (RVR), Wechsler Adult Intelligence Scale (DS and BD subtest) and Hamilton Depression Rating Scale; OR, Odds Ratio; PSD, Post-stroke Dementia; mRS, modified Rankin Scale; NART-R, National Adult Reading Test-Revised; IMD, Index of Multiple Deprivation; SED, Socioeconomic Deprivation; BI, Barthel Index; ACE-R, Addenbrooke's Cognitive Examination-Revised; VaD, Vascular Dementia; PSCI, Post-stroke Cognitive Impairment; MoCA, Montreal Cognitive Assessment; CDR, Clinical Dementia rating;

FMC, Free Medical Care; HADS-A, Hospital Anxiety and Depression Scale-Anxiety; HADS-D, Hospital Anxiety and Depression Scale-Depression; ADI, Area Deprivation Index.

Table S4. Results of Studies with Mortality After Stroke as the Secondary Outcome

Authors, Year, Journal	Title	CR Indicators	Outcome Definition	Type of Regression Model Used	Association Between CR and Mortality
Zhou et al, 2006, *BMC Public Health*	The effect of socioeconomic status on three-year mortality after first-ever ischemic stroke in Nanjing, China	Level of education, taxable income, occupation and housing space were used as indicators of SES. (Education: illiterate, primary, junior, senior, technical training or apprenticeship; university; Occupation: unemployed, manual, non-manual; Taxable income: ¥0, ¥0-¥100, >¥100; Housing space (㎡/person): <10, 10-20, 20-40, >40)	Mortality (Telephone and mail were used to obtain information about survival status.)	Cox proportional hazards models	Manual workers (HR = 5.44, 95%CI: 2.75-10.77) was significantly associated with three-year mortality after first-ever stroke. Those in the zero income group had a significant hazard ratio of 5.35 (95%CI: 2.95-9.70) and those in the intermediate income group 2.10 (95%CI: 1.24-3.58) when compared with those in the highest income group. Those in two of the three groups with the smallest housing space also had significant hazard ratios of 2.06 (95%CI: 1.16-3.65) and 1.68 (95%CI: 1.12-2.52) when compared with those in group with the largest housing space.
Cesaroni et al, 2009, *Stroke*	Socioeconomic differences in stroke incidence and prognosis under a Universal Healthcare System	Education, occupation, home ownership, family composition, and citizenship were combined to create an index of SEP, ranging from very well off (Level 1) to very	Mortality (Data on mortality was from the Regional Cause of Death Registry.)	Logistic regression	No association was found for SEP and mortality after stroke.

		underprivileged (Level 5).			
Toivanen et al, 2011, *Scand J Public Health*	Income differences in stroke mortality: A 12-year follow-up study of the Swedish working population	The income measure was classified into four groups by quartile values of the income distribution in the combined data for women and men.	Mortality (Information of mortality were from the Cause of Death Register.)	Cox proportional hazards models	The age-adjusted hazard ratio (95%CI) of lowest versus highest income quartile was 1.80 (1.48-2.19) for any stroke, 1.68 (1.29-2.17) for ICH and 2.23 (1.53-3.22) for BI in women, and the corresponding figures for men were 2.12 (1.92-2.34), 2.02 (1.77-2.31), and 2.09 (1.77-2.46).
Ojala-Oksala et al, 2012, *Stroke*	Educational history is an independent predictor of cognitive deficits and long-term survival in postacute patients with mild to moderate ischemic stroke	Education (0-6y, 7-9y, ⩾10y)	Mortality (Long-term survival data and causes of death were obtained from Statistics Finland.)	Cox regression	Educational history was associated independently with favorable poststroke survival (HR=0.86, $P<0.05$).
Brown et al, 2013, *Neurology*	Neighborhood socioeconomic disadvantage and mortality after stroke	The NSES index was constructed by summing the z scores of 6 SES indicators, range from quartile 1 as least disadvantaged neighborhoods to quartile 4 as the most disadvantaged Neighborhoods.	Mortality (Information on death were obtained from death certificates, autopsy and coroner's reports, hospital records, and interview with attending physicians, next of kin, and witnesses.)	Cox proportional hazards models	Mortality hazard 1 year after stroke was significantly higher among residents of neighborhoods with the lowest NSES than those in the highest NSES neighborhoods (HR = 1.77, 95%CI: 1.17-2.68).
Goulart et al, 2013, *BMC Neurol*	Predictors of long-term survival among first-ever ischemic and	Education (Illiterate, 1-7 y, ≥8 y)	Mortality (Information including	Cox proportional hazards models	The risk of death was two times higher among people with ischemic

	hemorrhagic stroke in a Brazilian stroke cohort		vital status during follow-up was updated through telephone contact, medical registers, and death certificates.)		stroke without formal education.
Pan et al, 2016, *Int J Stroke*	Socioeconomic deprivation and mortality in people after ischemic stroke: The China National Stroke Registry	Education, occupation and income were classified as indicators of SES. (Education: illiterate, 1-5 y, 6-9 y, 10-12 y, >12 y; Occupation: no job, retired, manual, non-manual; Income: <¥500, ¥500-¥1000, ¥1001-¥3000, ¥3001-¥5000, ¥5001-¥10,000, and >¥10,000.)	Mortality (The central telephone follow-up was performed by trained interviewers for all patients based on a standardized interview protocol.)	Multivariate adjusted logistic regression	In a 12-month follow-up, odds ratio for mortality in patients with low education was 1.25 (95%CI: 1.05-1.48), manual laboring 1.37 (95%CI: 1.09-1.72), and low income 1.19 (95%CI: 1.03-1.37).
Shin et al, 2017, *J Epidemiol*	Cross-level interaction between individual socioeconomic status and regional deprivation on overall survival after onset of ischemic stroke: National health insurance cohort sample data from 2002 to 2013	Individual house-hold incomes were categorized into three groups (low, 0-30th percentile; middle, 31st-70th percentile; high, 71st-100th percentile) and Carstairs index was calculated as regional deprivation, including residents in households headed by unskilled individuals, unemployed males, residents overcrowded and residences not owner occupied.	Mortality (Information on mortality were obtained from the Korean National Statistical Office.)	Cox proportional hazards models	For the middle income level, the patients in advantaged regions showed low HRs for overall mortality (12-month HR = 1.27, 95%CI: 1.13-1.44; 36-month HR = 1.25, 95% CI: 1.14-1.37) compared to the others in disadvantaged regions (12-month HR = 1.36, 95% CI: 1.19-1.56; 36-month HR = 1.30, 95% CI: 1.17-1.44).
Yan et al, 2017, *Int J Med Sci*	The influence of individual socioeconomic status on the	Education, occupation, annual income, and medical	Mortality (Information on mortality	Multivariate Cox regression	Both individual SES (HR = 0.767, 95% CI: 0.623-0.944; *P* = 0.012)

	clinical outcomes in ischemic stroke patients with different neighborhood status in Shanghai, China	insurance as indicators of SES were categorized to five groups from low to high level, for which a gradually increasing score (0-4) was assigned and the final summed score of each factor represented the individual SES.	were provided by patients and reliable proxy relatives.)		and neighborhood status (HR = 0.730, 95% CI: 0.582-0.916; *P* = 0.007) are independently associated with the clinical outcomes in ischemic stroke patients.
Vivanco-Hidalgo et al, 2019, *Stroke*	Association of Socioeconomic Status With Ischemic Stroke Survival	PCSA Index and drug dispensation were considered as indicators of SES, which score ranges from 0 (less deprived) to 100 (more deprived).	Mortality (Data on mortality were from the Catalan Central Registry of Insured Persons dataset)	Mixed-effects logistic and survival models	Association between the lowest socioeconomic individual status and short-term survival was not found (OR = 1.03; 95% CI: 0.76-1.40), although it existed in patients with < € 18 000 income/year (OR = 1.26, 95% CI: 1.10-1.45). At long-term, a gradient in mortality risk with decreasing individual socioeconomic status was found (HR = 1.52; 95% CI: 1.30-1.77).
Che et al, 2020, *J Am Heart Assoc*	Education level and long-term mortality, recurrent stroke, and cardiovascular events in patients with ischemic stroke	Education (Illiterate, primary, middle, college)	Mortality (Information on death were verified by examining hospital medical records.)	Cox proportional hazards models	Hazard ratios and 95% CIs of illiteracy versus college education were 2.79 (1.32-5.87) for all-cause mortality, 3.68 (1.51-8.98) for stroke-specific mortality,
Béjot et al, 2021, *Eur J Neurol*	Social deprivation and one-year survival after stroke: a prospective cohort study	The EPICES score was used to assess social deprivation, and patients with a score ≥30.17	Mortality (Survival was determined by follow-up, including	Multivariable Cox model	An excess in mortality was observed between 90 days and 12 months in deprived compared with

		considered as deprived.	contacting a next of kin or the patient's physician if the patient could not be reached.)		non-deprived patients (aHR = 1.97, 95%CI: 1.14-3.42, *p*=0.016). In patients with ICH, mortality at 12 months did not significantly differ according to deprivation status.
Zhu et al, 2021, *BMC Public Health*	Occupational class differences in outcomes after ischemic stroke: a prospective observational study	Occupation (White-collar, blue-collar, farmer)	Mortality (Date of death were verified by examining hospital medical records.)	Cox proportional hazards models	Compared with white-collar workers, farmers had fewer mortality events (HR = 0.50, 95% CI: 0.31-0.82), while there was no significant difference in blue-collar workers (HR = 0.74, 95% CI: 0.47-1.15).

HR, Hazard Ratio; SEP, Socioeconomic Position; NSES, Neighborhood Socioeconomic Status; PCSA Index, Primary Care Service Area Socioeconomic Index; EPICES, Evaluation de la Précarité et des Inégalités de santé dans les Centres d' Examen de santé;
